# Lipid profile of cerebrospinal fluid in multiple sclerosis patients: a potential tool for diagnosis

**DOI:** 10.1038/s41598-019-47906-x

**Published:** 2019-08-05

**Authors:** L. Nogueras, H. Gonzalo, M. Jové, J. Sol, A. Gil-Sanchez, J. V. Hervás, P. Valcheva, C. Gonzalez-Mingot, M. J. Solana, S. Peralta, R. Pamplona, L. Brieva

**Affiliations:** 1 Universitat de Lleida (UdL), Lleida, Spain; 2 Institut de Recerca Biomèdica de Lleida (IRB-Lleida), Lleida, Spain; 3 Hospital Universitario Arnau de Vilanova Hospital (HUAV), Lleida, Spain

**Keywords:** Multiple sclerosis, Multiple sclerosis

## Abstract

Multiple sclerosis (MS) is a complex multifactorial neuropathology. Although its etiology remains unclear, it has been demonstrated that the immune system attacks myelin, leading to demyelination and axonal damage. The involvement of lipids as one of the main components of myelin sheaths in MS and other demyelinating diseases has been postulated. However, it is still a matter of debate whether specific alteration patterns exist over the disease course. Here, using a lipidomic approach, we demonstrated that, at the time of diagnosis, the cerebrospinal fluid of MS patients presented differences in 155 lipid species, 47 of which were identified. An initial hierarchical clusterization was used to classify MS patients based on the presence of 25 lipids. When a supervised method was applied in order to refine this classification, a lipidomic signature was obtained. This signature was composed of 15 molecules belonging to five different lipid families including fatty acids (FAs). An FA-targeted approach revealed differences in two members of this family: 18:3n3 and 20:0 (arachidic acid). These results reveal a CSF lipidomic signature in MS patients at the time of diagnosis that might be considered as a potential diagnostic tool.

## Introduction

Multiple sclerosis (MS) is a highly complex central nervous system (CNS) disease characterized by a multifactorial pathogenesis and a highly heterogeneous clinical presentation^1^. MS is the main cause of disability in young adults^2^. One very active research field focuses on the discovery of new biomarkers that will be helpful in understanding the etiology of MS and in assisting early diagnosis and prognosis^3–6^.

One of the most important hypotheses about the origin of MS is that this disease is a consequence of an autoimmune response to self-antigens in a genetically susceptible person^7,8^. It has been demonstrated that the immune system attacks myelin, resulting in demyelination and axonal damage of the neurons^8^. Myelin sheath is rich in lipids (70–85% of dry weight); it contains about 700 different lipid species, specifically sphingolipids and glycerophospholipids^9,10^, whose role in MS and other demyelinating diseases has been postulated^11^. It remains unclear whether components of cerebrospinal fluid (CSF) contribute to myelin destruction and axonal loss or whether their presence is the consequence of these processes^12^, but the results of different studies suggest that fatty acids (FAs) are important regulators of the well-being of axons/neurons^13,14^.

Lipids are important components of mammalian cells. Among the different tissues of the human body, nerve tissues comprise some of the richest in lipid content^15,16^. Different lipid species play diverse roles in the CNS: cholesterol drives synaptogenesis in CNS neurons, FAs are precursors of signalling and structural lipids, corticoids and prostaglandins mediate inflammation, glycerophospholipids and glycosphingolipids are involved in membrane biogenesis, etc^9^. Lipid metabolism changes have also been described in several CNS diseases^15^. The study of lipids at disease onset could be an important tool to better understand the biological processes occurring at the beginning of the disease or even to help in the diagnosis/prognosis of MS.

Even now, MS diagnosis is not an easy task^17^; it is based on signs and symptoms consistent with the disease plus a set of diagnostic tests^18^ that have already been validated. The complex processes occurring in the brain from the early onset of the disease are still not completely understood. With this regard, some studies have suggested that lipid mediators are involved in the autoimmune attack against the neurons^19,20^. Thus, different antibody patterns of lipids have been identified, linked with different stages of the disease^20^. Moreover, lipid-specific oligoclonal IgM bands have been associated with different relevant factors over the course of the disease, such as brain atrophy, greater lesion load from the initial phase, and disability^21,22^. Additionally, higher phospholipid concentrations and lower sphingolipid levels have been described in brain tissue from patients^23^, and altered levels of certain ceramides have also been seen in MS^24^.

Lipidomics is an important resource for the study of multifactorial diseases^25^. Under pathological conditions, cells may suffer metabolic changes that could modify their lipidome^26^. These changes may be detected in the CSF, since this is the body fluid that best reflects brain environment, thanks to its close contact with this organ and its nutritional and scavenger functions. Consequently, the study of the CSF lipidome offers the opportunity to explore possible changes in the CNS and myelin. In this line, in the present work, we first applied a non-targeted or global lipidomic approach because we would like to measure as many lipid species as possible and compared between samples without bias^27^. After obtaining this global view we applied a targeted approach to particularly determine the CSF fatty acid composition. Therefore, the purpose of this study was to analyse possible changes in the lipidome of MS patients from the time of diagnosis, with a special focus on FAs.

## Materials and Methods

### Human samples

The study was approved by the local ethics committee at Arnau de Vilanova University Hospital (Lleida, Spain). It was conducted following the Code of Ethics of the World Medical Association (Declaration of Helsinki). Informed consent was obtained from all participants.

A retrospective study of 107 samples of CSF obtained in our hospital by lumbar puncture at the time of diagnosis was conducted. The MS patients included in this study in the period between 2001 and 2005 were diagnosed of relapsing-remitting MS according to 2001 McDonald criteria^28^. The principle of MS diagnosis in MS diagnostic criteria in 2001 and the following versions remains of wanting to meet the 3 diagnostic criteria, which include demonstration that there’s multiplicity in space, meaning multiple aspects of the central nervous system pathways involved; multiplicity over time, meaning that there is some ongoing disease activity or progression over time; and exclusion of other diagnoses that can look like MS. The presence of relapses but above all magnetic resonance imaging are very important to demonstrate dissemination of lesions in time and space and exclusion of other conditions. We chose as non-MS patients those individuals who, after a lumbar puncture, an MS diagnosis was discarded. CSF samples were obtained in the moment of diagnosis (2001–2005) and were included in the study after at least 5 years (2011) when we had data about their disease evolution.

Patients’ characteristics and diagnosis are shown in Table 1. Groups (MS group and non-MS group) were matched by sex and age given the influence that these variables may have on disease characteristics.

**Table 1:** Clinical features and diagnosis of multiple sclerosis *vs*. non-multiple sclerosis patients included in the study.

	MS (*n* = 53)	Non-MS (*n* = 54)	*p*-value
Sex			0.247
Women	38 (71.7%)	33 (61.1%)	
Men	15 (28.3%)	21 (38.9%)	
Age	46.77 ± 9.7	50.48 ± 12.1	0.083
Years since diagnosis	8 ± 3.39		
EDSS at diagnosis moment	1.3 ± 1.5	—	
Number of relapses before diagnosis	2.15 ± 0.92	—	
MS Diagnosis	53^a^		
Non-inflammatory ND	—	29	
Inflammatory diseases^b^	—	9	
Non-ND disease	—	16	

MS, Multiple sclerosis; ND, Neurological diseases.

^a^Patients were diagnosed of relapsing-remitting MS according to 2001 McDonald criteria^28^.

^b^Different from MS.

The diagnostic CSF was collected, centrifuged and stored at −80 °C until further analysis. Subjects of the study were not under any treatment at the time of the lumbar puncture.

### Chemicals

We obtained from Sigma-Aldrich (Madrid, Spain) synthetic lipids (also from Avanti Polar Lipids Inc., Alabaster, AL, USA), fatty acid methyl ester standards (also from Larodan Fine Chemicals, Mälmo, Sweden), methyl tert-butyl ether (MTBE) liquid chromatography coupled to mass spectrometry (LC-MS), acetonitrile LC-MS, isopropanol LC-MS, potassium chloride, chloroform, ammonium formate and ammonium hydroxide. We obtained acetone from Riedel-de-Häen (Seelze, Germany), methanol from Carlo Erba (Milano, Italy) and LC/MS-grade isopropanol and formic acid from Baker (Phillipsburg, NJ, USA).

### Lipidomic analysis: CSF lipidome

A previously validated method was used for lipid extraction^29^. Briefly, 5 μl of miliQ water and 20 μl of methanol were added to 10 μl of CSF sample. Samples were then shaken vigorously for 2 min. Then methyl tert-butyl ether (MTBE) containing lipid standards^30^ (isotopically labelled lipids) was added for external and internal standardization. Samples were then immersed in a water bath (ATU Ultrasonidos, Valencia, Spain) with an ultrasound frequency 40 kHz and power of 100 W, at 10 °C for 30 min. Then, 75 μL of miliQ water were added for mixing the organic phase, that was centrifuged (1400 g) 10 °C 10 min to separate the organic phase. Finally, the upper phase was collected and stored for mass-spectrometry analysis. A pool (20 µl of each sample) of all lipid extracts was prepared and used as quality control^31^.

Lipid extracts were analysed by LC-MS according to the method described by Castro-Perez *et al*.^25^. An Agilent UPLC 1290 system coupled to the Q-TOF MS/MS 6520 (Agilent Technologies, Barcelona, Spain) was used. A single run was performed to collect positive electrospray ionized lipid species.

Data analyses are detailed in Supplementary Material and Methods.

### Lipidomic analysis: CSF fatty acids composition

Briefly, samples were incubated for lipid extraction and FAs transesterification in 2 ml of 5% methanolic HCL at 75 °C for 90 min. FAs methyl esters were extracted by adding 2 ml of n-pentane and 1 ml of saturated NaCl solution. Samples were separated and evaporated under N_2_ gas n-pentane phase and finally dissolved in 80 µl of carbon disulphide. Gas chromatography (GC) analysis was then performed.

The GC method was used for separation with a DBWAX capillary column (30 m × 0.25 mm × 0.20 μm) in a GC System 7890 A with a Series Injector 7683B and a FID detector (Agilent Technologies, Barcelona, Spain). The temperature of the injector was 220 °C using the splitless mode. A constant rate (1.8 ml/min) of helium (99,99%) was maintained. The column temperature was held at 145 °C for 5 min; subsequently, the column temperature was increased by 2 °C/min to 245 °C for 50 min, and held at 245 °C for 10 min, and with a post-run of 250 °C for 10 min as previously described^16,28^.

We used lipid standards (Larodan Fine Chemicals, Malmö, Sweden) to identify FAs. Different indexes were calculated (see Supplementary Material and Methods). Due to experimental limitations, pools of different were created to analyse CSF FAs composition (Table 2). Each pool (600 µl) was performed using different CSF samples and was made according with sex and age criteria in order to obtain pools as similar as possible. Each pool was analysed as a unique sample.

**Table 2:** Pools used for fatty acid analysis.

Codification	Group	Sex	Age (years)	Number of samples/pool	Volume/sample (µl)
A	MS	Women	<40	8	75
B	MS	Women	>40	4	150
C	MS	Men	All	7	86
N	MS	Women	>40	6	100
Ñ	MS	Women	>40	6	100
O	MS	Women	>40	6	100
P	MS	Women	<40	4	150
Q	MS	Women	<40	4	150
R	MS	Men	>40	4	150
S	MS	Men	<40	4	150
D	Non-MS	Women	<40	7	86
E	Non-MS	Women	>40	8	75
F	Non-MS	Men	All	5	120
G	Non-MS	Women	>40	5	120
H	Non-MS	Women	>40	5	120
I	Non-MS	Women	<40	6	100
J	Non-MS	Men	>40	7	86
K	Non-MS	Men	<40	5	120
L	Non-MS	Women	All	4	150
M	Non-MS	Men	All	5	120

MS, multiple sclerosis.

### Statistics

Metaboanalyst software (www.metaboanalyst.ca) was used for CSF lipidome analyses. An unpaired t-test using the Benjamini Hochberg procedure for controlling false discovery rates (FDR) was used to compare MS and non-MS groups. For CSF FAs, composition analyses were performed with the SPSS software (SPSS, Chicago, IL, USA). A t-test was used for comparisons between multiple sclerosis and non-multiple sclerosis groups.

Statistical significance was considered when *p* < 0.05.

## Results

### Lipidomic signature of MS patients by non-supervised analyses

A non-targeted approach was performed to determine global lipidomic differences between MS and non-MS patients. This method detected 9532 lipid species. Only common features presented in at least 50% of the samples of each group were selected. As a result, 1094 molecules were included in the study. Of these, 155 were significantly different between the groups but only 47 were identified based on their exact mass, retention time and isotopic distribution: 30 glycerolipids, 5 sterol lipids, 4 FAs, 5 glycerophospholipids, and 3 sphingolipids (Table 3). Within the glycerolipid group, only five triglycerides but all diglycerides except one occurred at higher concentrations in the MS group. In the other families, lipids were also found to be more concentrated in MS patients while some others occurred at a higher concentration in the non-MS group.

**Table 3:** Identified compounds statistically significant in terms of false detection rate between multiple sclerosis and non-multiple sclerosis groups.

Lipid family	Compounds	m/z	Retention time	FDR	MS *vs*. non-MS	*p*-value
GL	TG(52:3)	1714.502	10.25259	0.068916	Down	0.0001388
	TG(58:3)	961.8085	8.904963	0.068916	Down	0.00016485
	TG(57:4)	947.7941	9.040551	0.2928	Down	0.025853
	TG(52:3)	895.7236	9.148117	0.11497	Down	0.00075693
	TG(61:10)	969.7727	8.892434	0.30356	Down	0.031078
	TG(37:2)	943.8401	10.278374	0.33716	Down	0.042597
	TG(55:5)	917.7423	8.589999	0.1865	Down	0.0057969
	TG(57:7)	919.7651	8.855682	0.19304	Down	0.0067051
	TG(61:8)	972.8497	10.11767	0.19578	Down	0.0084112
	TG(60:10)	937.7439	9.085229	0.21458	Down	0.010003
	TG(62:8)	986.8657	10.10129	0.24316	Down	0.012221
	TG(50:1)	850.7819	10.2558	0.24316	Down	0.013439
	TG(44:5)	1040.9073	10.139274	0.34663	Down	0.047253
	TG(59:6)	948.8528	10.26397	0.2875	Down	0.022184
	TG(44:4)	1042.9316	10.266933	0.33716	Down	0.042985
	TG(58:1)	965.82	10.05336	0.34663	Down	0.045241
	TG(56:6)	911.7205	9.042023	0.29858	Down	0.029317
	TG(64:10)	1028.844	10.11798	0.11497	Up	0.0013801
	TG(63:8)	983.8231	10.41375	0.16622	Up	0.0041023
	TG(59:2)	977.8285	9.264049	0.33648	Up	0.037175
	TG(56:4)	893.7833	9.696512	0.2875	Up	0.023736
	TG(57:6)	921.7742	9.030166	0.34663	Up	0.047129
	DG(32:1)	571.4445	4.743451	0.24316	Down	0.013843
	DG(38:7)	656.5246	3.772626	0.2875	Up	0.020668
	DG(38:6)	641.5141	8.54952	0.31655	Up	0.033695
	DG(32:2)	565.4724	4.200697	0.33838	Up	0.043612
	DG(18:3)	647.5659	9.252644	0.19442	Up	0.0075779
	DG(39:2)	645.5683	9.958723	0.16622	Up	0.0042619
	DG(42:5)	699.5929	9.16009	0.19442	Up	0.0076197
	DG(36:6)	613.4884	6.572807	0.19737	Up	0.0086599
ST	5beta-cholestane-3alpha,7alpha-diol	421.4002	5.411677	0.19442	Up	0.0081436
	5beta-dihydrotestosterone	561.4389	10.7082	0.27585	Up	0.01765
	22:0 cholesteryl ester	764.642	9.240857	0.31655	Down	0.033854
	cholest-5-en-3alpha-ol	368.3454	9.19711	0.11497	Down	0.00060565
FA	12-methyl-10-oxo-tridecanoic acid	484.3757	3.767164	0.11497	Up	0.00097344
	N-oleoylethanolamine	652.5233	2.823321	0.16155	Up	0.0033467
GP	PE-NMe(O,O-28:0)	620.5302	5.85945	0.19442	Up	0.007838
	PE(21:0)	519.3339	2.47881	0.11497	Up	0.0013882
	PC(P27:1)	785.6206	3.335041	0.24316	Down	0.014003
	PS(40:3)	841.5933	6.3180757	0.27616	Down	0.018932
	PC(42:6)	861.6224	6.616532	0.33716	Up	0.042498
	PC(25:2)	771.5785	7.811657	0.34663	Up	0.045934
SP	GlcCer(D42:0)	851.6578	7.877041	0.16155	Up	0.0030725
	C20 sulfatide	817.5873	6.036833	0.1865	Down	0.0058125
	CerP(42:2)	744.6147	9.41886	0.2875	Down	0.022255

Lipid species were identified by exact mass and retention time.

m/z, mass-to-charge ratio; FDR, false discovery rate; GL, glycerolipids; ST, sterol lipids; GP, glycerophospholipids; SP, sphingolipids; TG, triacylglycerols; DG, diacylglycerols; PE-NMe, phosphtatidylethanolamine- N-methylethanolamine; PE, phosphtatidylethanolamine; PC, phosphatidylcholine; PS, phosphatidylserine; GlcCer, glucosylceramide; CerP, Ceramide-phosphate.

**Figure 1:**
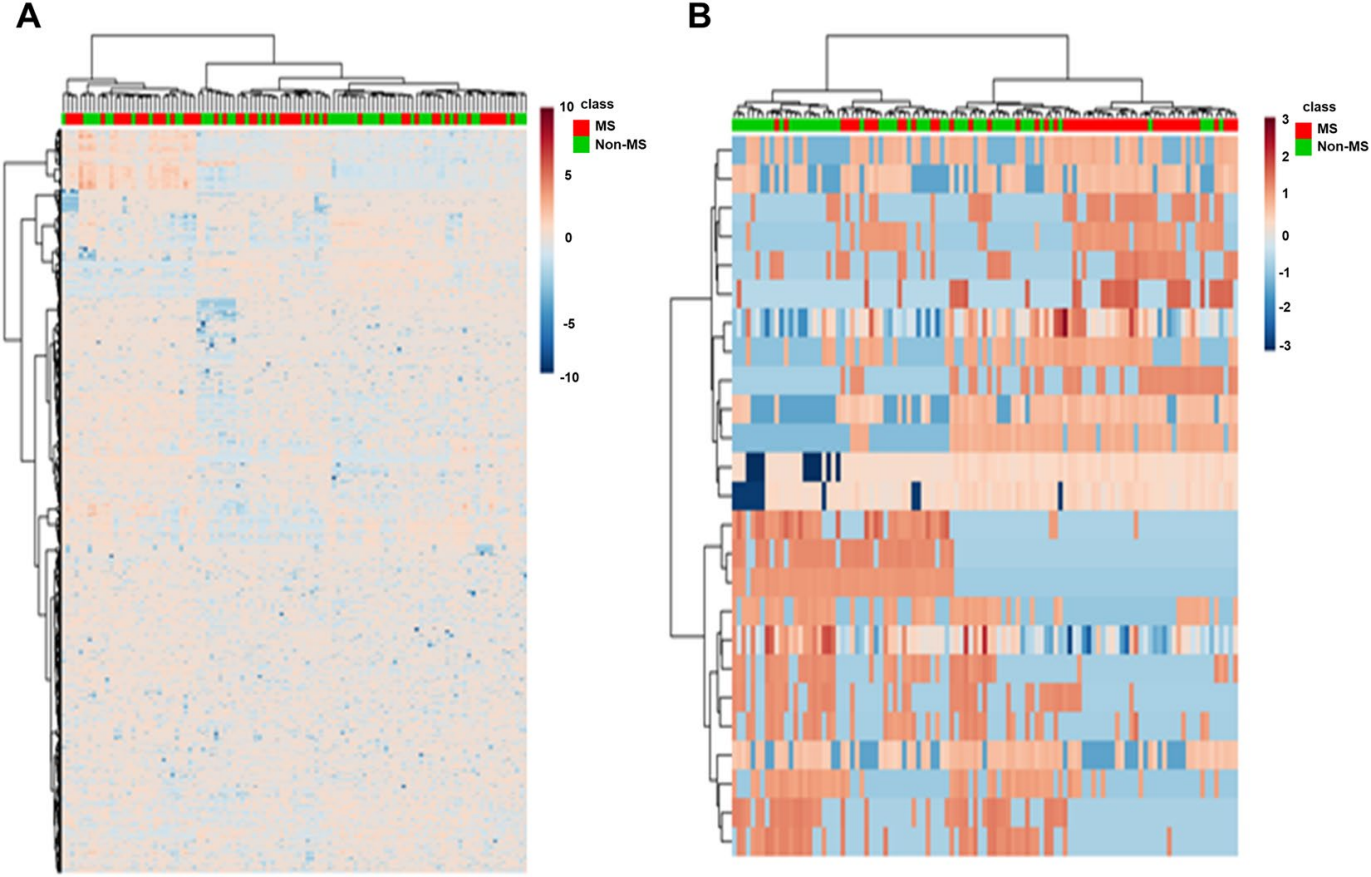
Heat map. (**A**) Heat map representation of all the molecules obtained by UPLC coupled to QTOF. (**B**) Heat map of the 25 most statistically significant lipid species obtained with t-test unpaired with Benjamini Hochberg Correction between non-MS group and MS patients. MS, multiple sclerosis.

**Figure 2:**
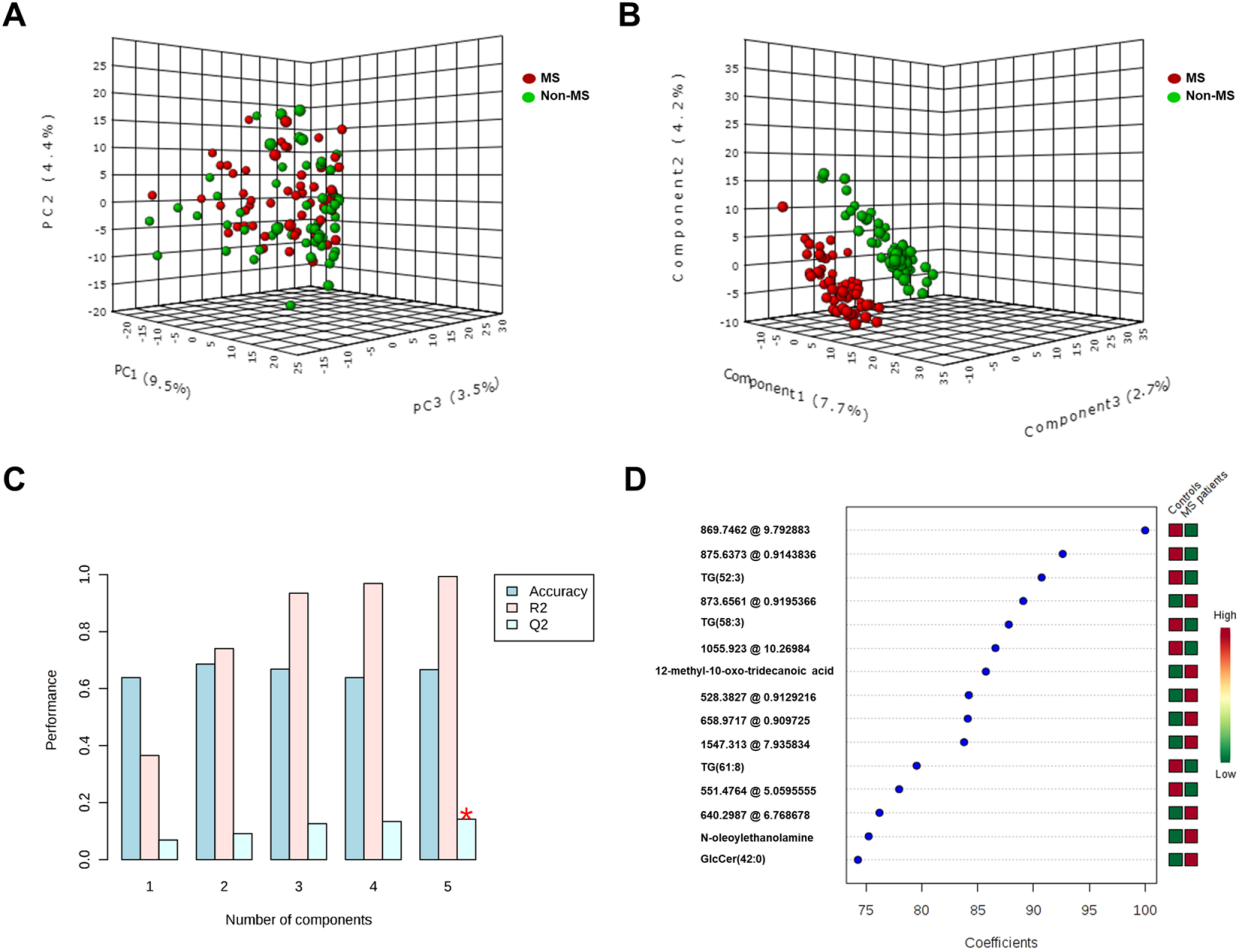
Multivariate statistics showing CSF lipidomic fingerprint in MS patients at the time of diagnosis. (**A**) Non-supervised analysis by PCA. (**B**) Supervised analysis by PLS-DA. (**C**) Cross validation values of PLS-DA model. (**D**) Variable importance in projection (VIP) score and identified lipid species. MS, multiple sclerosis.

To obtain a complete overview of lipidomic differences between groups, a hierarchical clustering analysis with all the lipid species selected was carried out (Fig. 1A). As observed, this method could not discriminate both groups according to their CSF global lipidome. Consequently, with the aim of defining a specific lipidomic signature for MS patients, hierarchical clustering was performed using only those 25 lipid species that showed the lowest *p-*value in the comparison between both groups (Supplementary Table S1; Fig. 1B). Results demonstrated that this new analysis could distinguish MS patients from the non-MS group. Similar results were obtained when data were analysed with the Principal Component Analyses (PCA), another unsupervised multivariate statistics method (Fig. 2A).

### Lipidomic signature of MS patients by supervised analyses

To further investigate whether a specific CSF MS lipidomic signature could be determined, data were analysed by the Partial Least Square-Discrimination Analyses (PLS-DA), a supervised method. As shown in Fig. 2B, the application of this methodology resulted in a perfect clusterization of MS and non-MS subjects at the time of diagnosis, suggesting the existence of a specific lipidomic signature. A cross validation of the PLS-DA model values is shown in Fig. 2C. Thus, lipidome analysis could accurately diagnose MS in 70% of the cases (accuracy = 0.7); the maximum value of R^2^ was obtained using 5 components. Q^2^ shows positive values, indicating that the model was effective for upgrading the prediction power.

After building the PLS-DA model, and in order to rank the distinctive features based on their significance in discriminating between groups, a variable importance in projection (VIP) score was applied (Fig. 2D). A 75% score was established as minimum. Statistically significant differences were determined in a total of 15 molecules between the MS and the non-MS groups. Among these molecules, 7 were identified based on their exact mass, retention time and isotopic distribution: 4 glycerolipids [TG(52:3), TG (58:3), TG(64:10) and TG(52:2)], 2 sterol lipids (cholest-5-en-3alpha-ol and dihydrotestosterone), and 1 FA (tridecanoic acid) (Table 3).

### Fatty acid composition in the CSF of MS patients

Finally, we performed a targeted analysis of the FA composition of the CSF. A total of 26 different FAs were identified. Several indexes (ACL, SFA, UFA, MUFA, PUFA, PUFAn3, PUFAn6, DBI, PI and AI) for the MS and the non-MS group were calculated. Results are shown in Table 4. As observed, statistically significant differences were obtained in two different FAs: 18:3n3 and 20:0 (arachidic acid). Both occurred at higher concentrations in MS patients at the time of diagnosis. Moreover, the anti-inflammatory index (AI) was found to be up-regulated in the CSF of MS patients.

**Table 4:** Cerebrospinal fluid total fatty acid composition (%mol) in multiple sclerosis patients and non-multiple sclerosis patients.

	MS patients	Non-MS patients	*p*-value
C14.0	1.4584 ± 0.11255	1.2850 ± 0.07007	0.199
C16.0	25.5481 ± 2.16850	26.7347 ± 0.35980	0.596
C16.1n7	0.8524 ± 0.08431	0.9425 ± 0.02084	0.314
C18.0	12.9257 ± 1.15606	12.8551 ± 0.46207	0.955
C18.1n9cis	16.1984 ± 1.41886	18.4624 ± 0.63264	0.162
C18.1n9trans	2.4464 ± 0.20571	2.6188 ± 0.07384	0.44
C18.2n6	5.9926 ± 0.60365	6.9335 ± 0.76935	0.345
C18.3n6	1.2841 ± 0.16512	1.1747 ± 0.06487	0.545
C18.3n3	0.2974 ± 0.04079	0.1642 ± 0.02147	0.010
C18.4n3	0.8177 ± 0.09040	0.9658 ± 0.07005	0.212
C20.0	0.9864 ± 0.04666	0.8472 ± 0.03369	0.026
C20.1n9	1.6032 ± 0.39412	1.2367 ± 0.05387	0.369
C20.2n6	0.4360 ± 0.02928	0.4093 ± 0.01458	0.425
C20.3n6	1.0453 ± 0.10963	0.9305 ± 0.07046	0.390
C20.4n6	3.3115 ± 0.33654	3.8642 ± 0.18690	0.168
C20.5n3	0.2014 ± 0.03405	0.1456 ± 0.2014	0.21
C22.0	1.4124 ± 0.10301	1.3205 ± 0.07728	0.484
C22.1n9	19.4135 ± 6.05948	14.3293 ± 0.89683	0.417
C22.4n6	0.1842 ± 0.03246	0.1715 ± 0.03291	0.787
C22.5n6	0.1480 ± 0.02066	0.1323 ± 0.04308	0.746
C22.5n3	0.3174 ± 0.04553	0.3081 ± 0.04395	0.885
C24.0	1.5414 ± 0.13123	1.7623 ± 0.04997	0.133
C22.6n3	0.3344 ± 0.03580	0.3092 ± 0.04184	0.653
C24.1n9	0.7500 ± 0.05176	0.7457 ± 0.04404	0.951
C24.5n3	0.1325 ± 0.01778	0.1250 ± 0.01607	0.758
C24.6n3	0.4157 ± 0.04715	0.4606 ± 0.03568	0.457
ACL	1793.5170 ± 31.21588	1764.8630 ± 3.45992	0.374
SFA	46.3900 ± 4.78370	44.8030 ± 0.80667	0.747
UFA	53.6100 ± 4.78370	55.1970 ± 0.80667	0.747
MUFA	41.2620 ± 4.84920	38.3840 ± 0.72128	0.564
PUFA	14.9700 ± 1.27658	16.8100 ± 1.02860	0.276
PUFAn3	2.5700 ± 0.22444	2.4770 ± 0.18774	0.754
PUFAn6	12.4017 ± 1.08330	14.3333 ± 1.13238	0.234
DBI	88.0139 ± 1.47043	89.4922 ± 1.81018	0.534
PI	41.0780 ± 3.24331	43.9898 ± 1.34036	0.418
AI	33.9720 ± 4.04480	23.2510 ± 2.75782	0.042

Data are represented as mean ± standard error. These results are performed using pools of samples, as it is specified in Methods Section.

MS, multiple sclerosis; ACL, average chain length; SFA, saturated fatty acids; UFA, unsaturated fatty acids; MUFA, monounsaturated fatty acids; PUFA, polyunsaturated fatty acids; PUFAn-3, polyunsaturated fatty acids series n-3; PUFAn-6, polyunsaturated fatty acids series n-6; DBI, double bond index; PI, peroxidizability index; AI, anti-inflammatory index.

## Discussion

MS is a chronic disease which is present in young adults. Nowadays diagnosis is based on four principles: (i) pattern and epidemiologic context of symptoms, (ii) cerebral/spinal magnetic resonance imaging lesions suggestive from demyelination with time and space dissemination, (iii) presence of oligoclonal bands in CSF and not in serum and (iv) the exclusion of other diagnoses^32^. Early diagnosis leads to early treatment, the approach that has the best outcomes in terms of progression and disability. A test for the diagnosis of the disease is urgently needed in this setting. Lipidomics is a powerful tool for the study of multifactorial diseases such as MS, and can be used to determine changes in compartments and structures that are fundamentally involved in the disease, such as myelin^25,26^. In our study, we have used lipidomics to demonstrate that MS patients present a different lipid profile at the time of diagnosis compared with non-MS subjects, underlining the importance of these molecules in this disease and the value of lipid signature characterization as a diagnostic tool. In this line, the description of the CSF lipidomic profile in the time of diagnosis could help to better understand the physiopathology of MS in early stages, to define the role of lipid metabolism in disease progression and to propose new biomarkers for monitoring the disease. Further, this information could be useful for new drugs development.

Both CSF and plasma are excellent sources of biological information which are easy to access^33^. However, unlike blood, CSF provides information from the CNS due to its proximity and contact. Additionally, it is useful for determining the progression of the disease from onset and can even help to predict disease development. In our study, an initial non-targeted approach in the CSF revealed statistically significant differences in 155 lipid species, 47 of which were identified based on their exact mass, retention time, and isotopic distribution. These results suggest that lipids might be useful biomarkers at the time of diagnosis. Further, multivariate statistics was applied in order to define a specific lipidomic signature. In this line, Although applying hierarchical clustering analyses and PCA method using all the lipid species we did not see a specific pattern for MS pathology when only those 25 the lipid species with de lowest p-value were used a much better clusterization was obtained. When supervised methods were applied, a perfect clusterization of patients was possible. Thus, 15 lipids were detected as the most relevant for distinguishing both groups, of which 8 were identified based on exact mass, retention time and isotopic distribution: four glycerolipids, one FA, two sterol lipids and one glycerophospholipid. The identified lipids belong to five different families, including sphingolipids. It is noteworthy that fingolimod, an effective treatment for MS, is an antagonist of sphingosine-1-phosphate (sphingolipid). This highlights the importance of this lipid family, whose levels, according to our results, are altered in CSF following disease onset. Another lipid family that showed differences was glycerolipids. Specifically, an up-regulation of diglycerides and a down-regulation of triglycerides was determined. This finding would be compatible with a defect of the acyl-CoA:diacylglycerol acyltransferase enzyme, which is related to a worse insulin sensitivity, a recently reported observation in patients with MS^34,35^. Hence, our results are in agreement with other studies that have found alterations in lipids, such as ceramides in CSF^36^, brain phosphatidylcholines^37^, and phospholipids and sphingolipids in white substance of the brain^23^. Finally, mammalian cell membranes including myelin are composed mainly of phosphatidylcholines and phosphatidylethanolamines, levels of which also differed between MS patients and non-MS group in our study^37^. Overall, differences in myelin sheath in CNS could mean more susceptibility to auto-antibody attack^22^.

A specific analysis of the CSF FA composition revealed fewer differences than in the lipidome. Of the 26 different FAs analysed, differences were obtained in only two. C20:0 or arachidic acid is a saturated FA (SFA) that, in our analysis, was increased in MS patients. Importantly, SFAs are implicated in inflammation^38^, a very relevant pathological process that occurs even before diagnosis of the disease. We also detected differences in alfa linolenic acid (C18:3n3). Although several FA deregulations have been found in neurological disorders such as Alzheimer’s disease, Parkinson’s disease and depression in brain tissue^38–40^, to the best of our knowledge, the increase of alfa linolenic acid (C18:3n3) in CSF at the time of diagnosis in MS patients has not been previously described. Indeed, in our study, patients presented almost twice as much of this essential FA. It has been reported that alpha linolenic acid has neuroprotective properties in an animal model of Parkinson’s disease^41^. Importantly, a prospective study has shown that alpha linolenic acid intake is inversely related to MS risk^42^. Although data on alfa linolenic acid intake were not collected in this study, it is worth remarking that an up-regulation of this FA at the time of diagnosis was found only in MS patients.

The main limitation of this study could be the number of patients enrolled and that all of them are from a restricted geographic area of Spain and from the same hospital. As a retrospective study the data being used was not designed to be used in a study. The fact that the non-MS group was not composed of healthy controls may be considered a limitation of our study. However, we would like to stress the fact that, despite the heterogeneity of this group, important differences in terms of the lipidomic profile were determined. Indeed, our approach could distinguish MS patients from other neuro-inflammatory disease carriers at the time of diagnosis based on their lipidomic signature. Finally, it is important to stress that, due to ethical considerations, lumbar puncture in healthy subjects is not permitted, so the inclusion of patients with other diseases is necessary to perform studies of this nature. Apart from the population and clinical factors, a limitation of the present study is that we are far from understanding the biological significance of this compositional complexity.

In conclusion, MS causes changes in the lipidomic CSF profile that could be considered as a potential diagnostic tool. This tool would make therapeutic decisions easier improving patients’ quality of life. Moreover, this investigation opens new perspectives for the understanding of why lipids are altered at disease onset and which pathways could be deregulated in MS and in other neurodegenerative diseases.

## Supplementary information

Supplementary material

## Data Availability

The authors confirm that the data supporting the findings of this study are available within the article and/or the Supplementary Materials.

## References

[1] Karussis D (2014). The diagnosis of multiple sclerosis and the various related demyelinating syndromes: A critical review. J. Autoimmun..

[2] Zéphir H (2018). Progress in understanding the pathophysiology of multiple sclerosis. Rev. Neurol. (Paris)..

[3] Comabella M, Montalban X (2014). Body fluid biomarkers in multiple sclerosis. Lancet Neurol..

[4] Grecchi S (2012). Search for cellular stress biomarkers in lymphocytes from patients with multiple sclerosis: a pilot study. PLoS One.

[5] Housley WJ, Pitt D, Hafler DA (2015). Biomarkers in multiple sclerosis. Clin. Immunol..

[6] Reinke SN (2014). Metabolomic profiling in multiple sclerosis: insights into biomarkers and pathogenesis. Mult. Scler..

[7] Ortiz GG (2013). Immunology and oxidative stress in multiple sclerosis: clinical and basic approach. Clin. Dev. Immunol..

[8] Sospedra M, Martin R (2005). Immunology of multiple sclerosis. Annu. Rev. Immunol..

[9] Cermenati G (2015). Lipids in the nervous system: from biochemistry and molecular biology to patho-physiology. Biochim. Biophys. Acta.

[10] O’Brien JS, Sampson EL (1965). Lipid composition of the normal human brain: gray matter, white matter, and myelin. J. Lipid Res..

[11] Schmitt S, Castelvetri LC, Simons M (2015). Metabolism and functions of lipids in myelin. Biochim. Biophys. Acta.

[12] Dutta R (2006). Mitochondrial dysfunction as a cause of axonal degeneration in multiple sclerosis patients. Ann. Neurol..

[13] Klosinski LP (2015). White Matter Lipids as a Ketogenic Fuel Supply in Aging Female Brain: Implications for Alzheimer’s Disease. EBioMedicine.

[14] Tafferner N (2016). Alpha-methylacyl-CoA racemase deletion has mutually counteracting effects on T-cell responses, associated with unchanged course of EAE. Eur. J. Immunol..

[15] Adibhatla RM, Hatcher JF (2007). Role of Lipids in Brain Injury and Diseases. Future Lipidol..

[16] Naudí Alba, Cabré Rosanna, Jové Mariona, Ayala Victoria, Gonzalo Hugo, Portero-Otín Manuel, Ferrer Isidre, Pamplona Reinald (2015). Lipidomics of Human Brain Aging and Alzheimer's Disease Pathology. International Review of Neurobiology.

[17] Beesley Rebecca, Anderson Valerie, Harding Katharine E, Joseph Fady, Tomassini Valentina, Pickersgill Trevor P, Robertson Neil P, Tallantyre Emma C (2018). Impact of the 2017 revisions to McDonald criteria on the diagnosis of multiple sclerosis. Multiple Sclerosis Journal.

[18] Thompson AJ (2018). Diagnosis of multiple sclerosis: 2017 revisions of the McDonald criteria. Lancet Neurol..

[19] Gonzalo H (2012). Lipidome analysis in multiple sclerosis reveals protein lipoxidative damage as a potential pathogenic mechanism. J. Neurochem..

[20] Kanter JL (2006). Lipid microarrays identify key mediators of autoimmune brain inflammation. Nat. Med..

[21] Villar LM (2015). Lipid-specific immunoglobulin M bands in cerebrospinal fluid are associated with a reduced risk of developing progressive multifocal leukoencephalopathy during treatment with natalizumab. Ann. Neurol..

[22] Villar LM (2005). Intrathecal synthesis of oligoclonal IgM against myelin lipids predicts an aggressive disease course in MS. J. Clin. Invest..

[23] Wheeler D, Bandaru VVR, Calabresi PA, Nath A, Haughey NJ (2008). A defect of sphingolipid metabolism modifies the properties of normal appearing white matter in multiple sclerosis. Brain.

[24] Moscatelli EA, Isaacson E (1969). Gas liquid chromatographic analysis of sphingosine bases in sphingolipids of human normal and multiple sclerosis cerebral white matter. Lipids.

[25] Castro-Perez JM (2010). Comprehensive LC-MS E lipidomic analysis using a shotgun approach and its application to biomarker detection and identification in osteoarthritis patients. J. Proteome Res..

[26] Adibhatla RM, Hatcher JF, Dempsey RJ (2006). Lipids and lipidomics in brain injury and diseases. AAPS J..

[27] Patti GJ, Yanes O, Siuzdak G (2012). Innovation: Metabolomics: the apogee of the omics trilogy. Nat. Rev. Mol. Cell Biol..

[28] McDonald WI (2001). Recommended diagnostic criteria for multiple sclerosis: guidelines from the International Panel on the diagnosis of multiple sclerosis. Ann. Neurol..

[29] Pizarro C, Arenzana-Rámila I, Pérez-del-Notario N, Pérez-Matute P, González-Sáiz J-M (2013). Plasma lipidomic profiling method based on ultrasound extraction and liquid chromatography mass spectrometry. Anal. Chem..

[30] Jové M (2018). Lipidomics reveals altered biosynthetic pathways of glycerophospholipids and cell signaling as biomarkers of the polycystic ovary syndrome. Oncotarget.

[31] Want EJ (2013). Global metabolic profiling of animal and human tissues via UPLC-MS. Nat. Protoc..

[32] Brownlee WJ, Hardy TA, Fazekas F, Miller DH (2017). Diagnosis of multiple sclerosis: progress and challenges. Lancet.

[33] Shahim P, Månsson J-E, Darin N, Zetterberg H, Mattsson N (2013). Cerebrospinal fluid biomarkers in neurological diseases in children. Eur. J. Paediatr. Neurol..

[34] Penesova A (2015). Hyperinsulinemia in newly diagnosed patients with multiple sclerosis. Metab. Brain Dis..

[35] Timmers S (2011). Paradoxical increase in TAG and DAG content parallel the insulin sensitizing effect of unilateral DGAT1 overexpression in rat skeletal muscle. PLoS One.

[36] Vidaurre OG (2014). Cerebrospinal fluid ceramides from patients with multiple sclerosis impair neuronal bioenergetics. Brain.

[37] Trépanier M-O (2018). Phosphatidylcholine 36:1 concentration decreases along with demyelination in the cuprizone animal model and in post-mortem multiple sclerosis brain tissue. J. Neurochem..

[38] Hussain G, Schmitt F, Loeffler J-P (2013). & Gonzalez de Aguilar, J.-L. Fatting the brain: a brief of recent research. Front. Cell. Neurosci..

[39] Astarita G (2011). Elevated stearoyl-CoA desaturase in brains of patients with Alzheimer’s disease. PLoS One.

[40] Conklin SM (2010). Age-related changes of n-3 and n-6 polyunsaturated fatty acids in the anterior cingulate cortex of individuals with major depressive disorder. Prostaglandins. Leukot. Essent. Fatty Acids.

[41] Shashikumar S, Pradeep H, Chinnu S, Rajini PS, Rajanikant GK (2015). Alpha-linolenic acid suppresses dopaminergic neurodegeneration induced by 6-OHDA in C. elegans. Physiol. Behav..

[42] Bjørnevik K, Chitnis T, Ascherio A, Munger KL (2017). Polyunsaturated fatty acids and the risk of multiple sclerosis. Mult. Scler. J..

