# Supplementary material for: Lipid profile of cerebrospinal fluid in multiple sclerosis patients: a potential tool for diagnosis
Source: Sci Rep. 2019 Aug 5;9:11313. doi: 10.1038/s41598-019-47906-x (PMC6683197; doi:10.1038/s41598-019-47906-x)
Supplement: Supplementary file 1 — Supplementary material [file 41598_2019_47906_MOESM1_ESM.pdf]

**Lipid profile of cerebrospinal fluid in multiple sclerosis patients: a potential tool for diagnosis**

Nogueras L.[1,2*#], Gonzalo H.[2*†], Jové M.[1,2], Sol [J1,2],Gil-Sanchez A.[2], Hervás J.V.[3], Valcheva P.[2], Gonzalez-Mingot C.[3] Solana M.J.[3], Peralta S.[3], Pamplona R.[1,2], Brieva L.[3†]

**Cerebrospinal fluid data analyses:**

Data were analysed using the MassHunter Data Analysis Software (Agilent Technologies, Barcelona, Spain) to collect the results. The Molecular Feature Extractor algorithm (Agilent Technologies, Barcelona, Spain) was used to obtain molecular features of the samples by MassHunter Qualitative Analysis Software (Agilent Technologies, Barcelona, Spain), as previously described[1].

Samples were selected with a minimum absolute abundance of 200 counts. A room temperature window of 0.1% ± 0.25 min and a mass window of 5.0 ppm ± 2.0 mDa were used to align compounds from different samples. Only common features (found in at least 50% of the samples of the same condition) were analysed. We performed unsupervised Hierarchical Clustering analyses and Principal Component Analyses (PCA) and supervised analysis (Partial Least Square-Discrimination Analyses [PLS-DA]), as well as variable importance in projection (VIP) using Metaboanalyst software. Differences obtained by t-test were investigated in LIPID MAPS[2], Metlin[3] and HMDB[4] to confirm the identification.

**Cerebrospinal fluid fatty acid composition: indexes**

The following indexes were calculated: saturated fatty acids (SFA), unsaturated fatty acids (UFA), monounsaturated fatty acids (MUFA), polyunsaturated fatty (PUFA) from n-3 and n-6 series (PUFAn-3 and PUFAn-6), and average chain length = ([Σ%Total14 × 14] + [Σ%Total16 × 16] + [Σ%Total18 × 18] + [Σ%Total20 × 20] + [Σ% Total22 × 22] + [Σ% Total24 × 24])/100.

The density of double bonds in the membrane was calculated with the Double Bond Index = ([1 × Σmol% monoenoic] + [2 × Σmol% dienoic] + [3 × Σmol% trienoic] + [4 × Σmol% tetraenoic] + [5 × Σmol% pentaenoic] + [6 × Σmol% hexaenoic]).

Membrane susceptibility to peroxidation was calculated with the Peroxidizability Index (PI) = ([0.025 × Σmol% monoenoic] + [1 × Σmol% dienoic] + [2 × Σmol% trienoic] + (4 × Σmol% tetraenoic] + [6 × Σmol% pentaenoic] + [8 × Σmol% hexaenoic]) [5].

**References:**


1 Sana, T. R., Roark, J. C., Li, X., Waddell, K. & Fischer, S. M. Molecular formula and METLIN Personal Metabolite Database matching applied to the identification of compounds generated by LC/TOF-MS. *Journal of biomolecular techniques : JBT* **19**, 258-266 (2008).

2 LIPID MAPS® Lipidomics Gateway. <http://www.lipidmaps.org/> (

3 METLIN. *MS/MS metabolite database*, <https://metlin.scripps.edu> (

4 HMDB. *The Human Metabolome Database*, <http://www.hmdb.ca> (

5 Cabré, R. *et al.* Lipid Profile in Human Frontal Cortex Is Sustained Throughout Healthy Adult Life Span to Decay at Advanced Ages. *Journals of Gerontology - Series A Biological Sciences and Medical Sciences* **73** (2018).

**Supplementary table S1**

| Compound | Mass | Retention time | FDR | MS vs. Non-MS | *p*-value |
|---|---|---|---|---|---|
| 697.5671@7.74 | 697.5671 | 7.742412 | 0.068916 | Up | 1.89E-04 |
| 444.2531@0.91 | 444.2531 | 0.9125238 | 0.11497 | Up | 4.56E-04 |
| 528.3827@528.38 | 528.3827 | 0.9129216 | 0.11497 | Up | 6.38E-04 |
| 1055.923@10.26 | 1055.923 | 10.26984 | 0.11497 | Down | 1.07E-03 |
| 869.7462@9.79 | 869.7462 | 9.792883 | 0.11497 | Down | 1.23E-03 |
| 945.8383@10.1 | 945.8383 | 10.10082 | 0.11497 | Down | 1.47E-03 |
| 658.9717@0.90 | 658.9717 | 0.909725 | 0.11809 | Up | 1.70E-03 |
| 433.3099@2.76 | 433.3099 | 2.76545 | 0.11809 | Down | 1.73E-03 |
| 577.3868@2.48 | 577.3868 | 2.487017 | 0.13436 | Up | 2.11E-03 |
| 433.3102@3.2 | 433.3102 | 3.2065 | 0.13436 | Down | 2.21E-03 |
| 444.2275@0.9 | 444.2275 | 0.9080975 | 0.15828 | Down | 2.75E-03 |
| 523.3847@3.85 | 523.3847 | 3.856381 | 0.16155 | Down | 3.33E-03 |
| 901.7429@8.66 | 901.7429 | 8.66991 | 0.16155 | Down | 3.40E-03 |
| 446.3401@6.56 | 446.3401 | 6.565541 | 0.16424 | Up | 3.64E-03 |
| 266.9601@0.74 | 266.9601 | 0.7447813 | 0.16424 | Up | 3.89E-03 |
| 640.2987@6.76 | 640.2987 | 6.768678 | 0.16424 | Up | 3.90E-03 |
| 560.382@0.9 | 560.382 | 0.9095536 | 0.17876 | Up | 4.74E-03 |
| 662.4499@7.94 | 662.4499 | 7.945977 | 0.1865 | Up | 5.12E-03 |
| 740.6461@9.84 | 740.6461 | 9.847604 | 0.1865 | Up | 5.56E-03 |

| | | | | | |
|---|---|---|---|---|---|
| 738.6284@9.19 | 738.6284 | 9.193216 | 0.1865 | Down | 5.59E-03 |
| 842.7561@10.3 | 842.7561 | 10.30109 | 0.1865 | Down | 6.22E-03 |
| 928.7886@9.74 | 928.7886 | 9.74718 | 0.1865 | Down | 6.23E-03 |
| 612.6015@9.37 | 612.6015 | 9.373285 | 0.1865 | Up | 6.31E-03 |
| 884.5595@0.9 | 884.5595 | 0.9066936 | 0.19442 | Down | 7.59E-03 |
| 1547.313@7.93 | 1547.313 | 7.935834 | 0.19442 | Up | 7.86E-03 |
| 905.7673@9.05 | 905.7673 | 9.056 | 0.19442 | Down | 8.17E-03 |
| 813.4384@9.4 | 813.4384 | 9.409812 | 0.19769 | Down | 8.85E-03 |
| 755.7384@7.01 | 755.7384 | 7.015704 | 0.20318 | Down | 9.29E-03 |
| 762.6136@8.14 | 762.6136 | 8.14451 | 0.21689 | Down | 1.03E-02 |
| 873.6561@0.91 | 873.6561 | 0.9195366 | 0.23085 | Up | 1.12E-02 |
| 1098.757@0.89 | 1098.757 | 0.8996832 | 0.24316 | Up | 1.27E-02 |
| 1142.784@0.89 | 1142.784 | 0.8980227 | 0.24316 | Down | 1.28E-02 |
| 550.3845@0.90 | 550.3845 | 0.9038666 | 0.24316 | Up | 1.28E-02 |
| 837.6288@8.52 | 837.6288 | 8.526357 | 0.24316 | Down | 1.31E-02 |
| 1709.467@10.09 | 1709.467 | 10.09186 | 0.24316 | Down | 1.32E-02 |
| 875.6373@0.91 | 875.6373 | 0.9143836 | 0.24316 | Down | 1.38E-02 |
| 595.5217@8.26 | 595.5217 | 8.265211 | 0.24528 | Up | 1.43E-02 |
| 711.5826@5.4 | 711.5826 | 5.404071 | 0.25835 | Up | 1.54E-02 |
| 9087.114@3.49 | 9087.114 | 3.494062 | 0.26571 | Down | 1.60E-02 |
| 556.4318@5.06 | 556.4318 | 5.060979 | 0.27536 | Down | 1.69E-02 |
| 4904.395@0.87 | 4904.395 | 0.87948 | 0.27585 | Down | 1.73E-02 |

| | | | | | |
|---|---|---|---|---|---|
| 1701.578@10.41 | 1701.578 | 10.41215 | 0.27585 | Up | 1.74E-02 |
| 507.4506@5.24 | 507.4506 | 5.249946 | 0.27616 | Up | 1.80E-02 |
| 1739.519@10.26 | 1739.519 | 10.260922 | 0.27616 | Down | 1.86E-02 |
| 1548.832@9.09 | 1548.832 | 9.091001 | 0.27616 | Up | 1.87E-02 |
| 523.4453@4.19 | 523.4453 | 4.199884 | 0.27616 | Up | 1.87E-02 |
| 811.5032@2.44 | 811.5032 | 2.44784 | 0.2875 | Up | 2.17E-02 |
| 813.4384@9.4 | 813.4384 | 9.409812 | 0.2875 | Up | 2.27E-02 |
| 617.2207@6.34 | 617.2207 | 6.340461 | 0.2875 | Up | 2.28E-02 |
| 294.2219@9.17 | 294.2219 | 9.179681 | 0.2875 | Down | 2.30E-02 |
| 579.5066@5.85 | 579.5066 | 5.855009 | 0.2875 | Up | 2.31E-02 |
| 679.4743@7.94 | 679.4743 | 7.943839 | 0.2875 | Up | 2.33E-02 |
| 1034.804@6.38 | 1034.804 | 6.389524 | 0.2875 | Down | 2.34E-02 |
| 777.5545@7.92 | 777.5545 | 7.92077 | 0.2875 | Up | 2.38E-02 |
| 556.4296@4.33 | 556.4296 | 4.331299 | 0.2875 | Up | 2.39E-02 |
| 399.295@0.9 | 399.295 | 0.9008861 | 0.2875 | Up | 2.39E-02 |
| 604.4477@7.37 | 604.4477 | 7.371906 | 0.29154 | Up | 2.48E-02 |
| 931.7911@9.1 | 931.7911 | 9.109068 | 0.29154 | Down | 2.50E-02 |
| 795.6152@8.59 | 795.6152 | 8.590509 | 0.29154 | Up | 2.52E-02 |
| 561.4417@7.03 | 561.4417 | 7.0302153 | 0.29154 | Down | 2.53E-02 |
| 1494.4241@7.45 | 1494.4241 | 7.457932 | 0.2928 | Down | 2.60E-02 |
| 696.3606@7.96 | 696.3606 | 7.963248 | 0.29304 | Down | 2.64E-02 |
| 572.4262@4.03 | 572.4262 | 4.0391026 | 0.29304 | Up | 2.73E-02 |

| | | | | | |
|---|---|---|---|---|---|
| 589.681@7 | 589.681 | 7.004667 | 0.29304 | Down | 2.74E-02 |
| 772.6452@9.02 | 772.6452 | 9.028791 | 0.29304 | Down | 2.75E-02 |
| 452.3637@3.84 | 452.3637 | 3.848778 | 0.29304 | Up | 2.75E-02 |
| 749.6622@9.35 | 749.6622 | 9.355952 | 0.29304 | Up | 2.78E-02 |
| 791.7087@6.57 | 791.7087 | 6.573787 | 0.29304 | Up | 2.79E-02 |
| 306.1863@6.47 | 306.1863 | 6.474189 | 0.29677 | Down | 2.85E-02 |
| 1407.1306@9.14 | 1407.1306 | 9.14772 | 0.29858 | Down | 2.94E-02 |
| 651.9692@0.90 | 651.9692 | 0.90748817 | 0.29858 | Up | 2.95E-02 |
| 1156.3748@7.38 | 1156.3748 | 7.3800464 | 0.30284 | Up | 3.02E-02 |
| 1094.3173@9.57 | 1094.3173 | 9.573249 | 0.30356 | Up | 3.07E-02 |
| 710.567@9.24 | 710.567 | 9.249712 | 0.30356 | Up | 3.10E-02 |
| 435.3933@4.6 | 435.3933 | 4.6034064 | 0.30987 | Down | 3.20E-02 |
| 216.042@0.85 | 216.042 | 0.8515834 | 0.31356 | Up | 3.27E-02 |
| 1367.1342@9.23 | 1367.1342 | 9.230128 | 0.31356 | Down | 3.30E-02 |
| 773.3503@8.75 | 773.3503 | 8.757948 | 0.33582 | Up | 3.64E-02 |
| 173.1414@0.79 | 173.1414 | 0.79630005 | 0.33582 | Up | 3.65E-02 |
| 619.4313@0.9 | 619.4313 | 0.9077692 | 0.33648 | Up | 3.72E-02 |
| 611.4969@3.89 | 611.4969 | 3.898938 | 0.33716 | Up | 3.80E-02 |
| 852.5342@3.02 | 852.5342 | 3.025107 | 0.33716 | Up | 3.84E-02 |
| 166.0271@6.31 | 166.0271 | 6.3105288 | 0.33716 | Up | 3.89E-02 |
| 412.2633@5.92 | 412.2633 | 5.925239 | 0.33716 | Down | 3.93E-02 |
| 579.607@9.47 | 579.607 | 9.475945 | 0.33716 | Up | 3.96E-02 |

| | | | | | |
|---|---|---|---|---|---|
| 998.6639@0.9 | 998.6639 | 0.9091111 | 0.33716 | Down | 3.97E-02 |
| 887.7509@8.95 | 887.7509 | 8.958172 | 0.33716 | Down | 3.97E-02 |
| 858.6052@6.55 | 858.6052 | 6.55954 | 0.33716 | Up | 4.05E-02 |
| 972.882@10.59 | 972.882 | 10.590237 | 0.33716 | Down | 4.05E-02 |
| 388.2494@0.88 | 388.2494 | 0.88027644 | 0.33716 | Up | 4.12E-02 |
| 848.8249@10.52 | 848.8249 | 10.525763 | 0.33716 | Up | 4.13E-02 |
| 610.4539@0.93 | 610.4539 | 0.9300715 | 0.33716 | Down | 4.15E-02 |
| 656.515@6.36 | 656.515 | 6.3626733 | 0.33716 | Down | 4.17E-02 |
| 954.7443@8.58 | 954.7443 | 8.589926 | 0.33716 | Up | 4.18E-02 |
| 834.5913@0.9 | 834.5913 | 0.9077015 | 0.33716 | Down | 4.21E-02 |
| 816.7668@10.1 | 816.7668 | 10.10611 | 0.33716 | Up | 4.31E-02 |
| 756.6438@9.15 | 756.6438 | 9.154258 | 0.34663 | Up | 4.54E-02 |
| 305.3186@3.09 | 305.3186 | 3.098807 | 0.34663 | Up | 4.69E-02 |
| 672.5777@10.65 | 672.5777 | 10.656383 | 0.34663 | Up | 4.71E-02 |
| 889.7681@9.15 | 889.7681 | 9.154843 | 0.34663 | Down | 4.74E-02 |
| 1032.7596@4.74 | 1032.7596 | 4.7485476 | 0.34663 | Down | 4.76E-02 |
| 832.4308@2.45 | 832.4308 | 2.4514945 | 0.34663 | Up | 4.83E-02 |
| 765.6868@9.65 | 765.6868 | 9.65807 | 0.34663 | Down | 4.84E-02 |
| 688.4883@0.9 | 688.4883 | 0.9087107 | 0.34663 | Up | 4.85E-02 |
| 532.4763@5.96 | 532.4763 | 5.966416 | 0.35255 | Up | 4.99E-02 |
| 588.5239@8.4 | 588.5239 | 8.409516 | 0.35255 | Down | 5.00E-02 |

Unidentified lipids are represented as exactmass@retentiontime
